# Evolution of Electronic Noses from Research Objects to Engineered Environmental Odour Monitoring Systems: A Review of Standardization Approaches

**DOI:** 10.3390/bios9020075

**Published:** 2019-05-31

**Authors:** Domenico Cipriano, Laura Capelli

**Affiliations:** 1 Ricerca sul Sistema Energetico (RSE), via Rubattino 54, 20134 Milano, Italy; domenico.cipriano@rse-web.it; 2 Politecnico di Milano, Department of Chemistry, Materials, and Chemical Engineering “Giulio Natta”, Piazza Leonardo da Vinci 32, 20133 Milano, Italy

**Keywords:** air quality, technical standards, quality protocols, emission monitoring, sensor arrays, performance testing, minimum requirements

## Abstract

Since electronic noses are used more and more for air quality monitoring purposes, and in some countries are starting to have a legal value, there is a need for standardization and programs for the quality verification of instruments. Such quality programs have the aim to guarantee the main characteristics of the instrument for both the final user and local authorities, let the user establish a suitable maintenance procedure and give information on measurement uncertainty. One critical aspect when dealing with electronic noses for environmental odour monitoring is that environmental odours are complex mixtures that are not repeatable nor reproducible, giving that they are not suitable for quality verifications. This paper aims to review and discuss the different approaches that can be adopted in order to perform quality checks on electronic noses (e-noses) used for environmental odour monitoring, thereby referring to existing technical standards, such as the Dutch NTA 9055:2012, the new German VDI 3518-3:2018, and the Italian UNI 1605848 project, which directly refer to electronic noses. Moreover, also the European technical standards that are prescriptive for automatic measuring systems (AMSs) are taken into consideration (i.e., EN 14181:2014 and EN 15267:2009), and their possible applicability to electronic noses is investigated. Finally, the pros and cons of the different approaches are presented and discussed in the conclusions section.

## 1. Introduction

Since the first report of an electronic nose based on chemical sensors and pattern recognition in 1982 [1], the instruments have experienced a significant development; they have been studied by several research groups over the world for a number of diverse possible applications in different sectors. The most interesting and promising sectors for the application of electronic noses that can be found in the scientific literature concern the food industry (e.g., [2,3,4,5,6]), medical diagnosis (e.g., [7,8,9,10,11,12,13]), and environmental monitoring (e.g., [14,15,16]).

It is a fact that, despite several promising results, practical applications of electronic noses in real-life are still limited [17]. Besides some technical criticalities, comprising for instance sensor lack of sensitivity/selectivity [18,19], interference with temperature and humidity [20], and drift [21,22,23], the lack of standardization also represents an important limit towards large-scale diffusion of electronic noses at an industrial level [15,24]. Given the wide range of different electronic noses available on the market, often based on different sensor types [25,26,27], data processing and pattern recognition systems [28,29], and/or functioning principles [4,30], it is very important that precise procedures for the verification of the instrument suitability for the desired application and its utilization are established [24].

Especially in the environmental field, in the last 15 years, electronic noses have become more and more popular air quality monitoring tools. As a matter of fact, they currently represent the only method available for the continuous monitoring of odours in the field [15,31].

Odour pollution is nowadays a serious environmental problem, and one of the major causes of citizens’ complaints to local authorities. For this reason, odours are now subject to control and regulation in many countries [32,33], thus making it necessary to have reliable and accurate methods for the assessment of odour impacts. Indeed, dynamic olfactometry, which is the reference method for the measurement of odour concentration, is intrinsically discontinuous, and applies to the quantification of emission sources, as stated in the scope of the reference standard for dynamic olfactometry [34]. Dispersion models are commonly applied to evaluate how odour emissions disperse from the source to the receptors and to evaluate odour impacts [31,33]. The input parameter that defines odour emissions in dispersion models is the so called “odour emission rate” (OER), expressed in odour units per unit time (i.e., ou_E_/s). However, there are many situations in which source characterization and the estimation of a representative OER may become extremely complex, for which the use of electronic noses is particularly indicated for odour impact or exposure assessment purposes. Such cases include for instance sources having variable emissions over time, whereby it is difficult to associate a specific OER to every hour of the simulation domain. Such variable emissions are typical of discontinuous productions, including for instance plants working “on order”, which work just a few hours a day (e.g., asphalts production), or manufacture different products depending on customers’ requests (e.g., pharmaceuticals). Another critical case is represented by diffuse sources, such as not-ventilated sheds or tanks, for which the estimation of the emitted air flow, which is necessary as input data for the dispersion model, is not always possible [35]. However, it is worth mentioning that, in recent years, great efforts are being made in the field of complex source characterization (e.g., [36,37]).

For this reason, in the field of environmental odour monitoring, electronic noses are rapidly turning from being only scientific and research objects to proper air quality monitoring tools. Besides examples describing applications of e-noses as air quality monitoring tools for odour impact assessment, alone or in combination with other techniques [38,39,40], there are also some situations in which e-noses are prescribed on a regulatory level [41,42]. It is clear that, when odour monitoring data produced by electronic noses start having a legal value, the need arises to have standards and quality programs allowing to ensure the quality of the whole monitoring process. As a general rule, standards play an important role for developing functional and reliable products for the global marketplace: they typically provide performance criteria that can be used to optimize the reliability and safety of new products [43]. According to this, standardized quality protocols are particularly needed for the instruments’ performance verification.

As a general rule, such quality programs have the aim to guarantee the main characteristics of the instrument for both the final user and the local authorities, let the user establish a suitable maintenance procedure and give information on measurement uncertainty [44].Given the intrinsic complexity of electronic nose features, and the wide variety of instrument types—sometimes based on different functioning principles and sensor types [16,25,30]—available on the market, standardization in this field should not concern the instrument hardware, but requirements on its performance could be fixed. This approach allows the instrument to be considered as a “black box”, by only taking into account the output metrics related to a given stimulus (input), thus ignoring the model that is used to transform the sensor signals into this output. One critical aspect regarding the application of electronic noses to environmental odour monitoring is that such odours are typically complex mixtures of hundreds of different compounds (e.g., [45,46,47]), and thus not repeatable nor reproducible. Environmental odours are therefore unsuitable for quality verifications, which require the standard reference materials that are tested to be repeatable and reproducible. However, some attempts of standardization have been carried out over the years, and will be discussed further on in this paper.

This paper has the aim of highlighting the importance of establishing suitable quality protocols applicable to electronic noses used as environmental odour monitoring systems and to describe some different approaches that can be adopted in order to perform quality checks on such instruments, thereby reviewing the relevant standards and publications in this field, and critically discuss the pros and cons related to their practical applicability.

For this purpose, the paper is divided into three parts.

The first part (Section 2) represents a state-of-the-art overview of how, in the last 15 years, electronic noses for environmental odour monitoring have evolved from a purely scientific and research level to become proper air quality monitoring tools used by plant operators as well as by local authorities for the management of odour issues through quantification of exposure or identification of emission sources. This state-of-the-art does not claim to provide an exhaustive review of all literature studies describing the application of electronic noses for environmental odour monitoring, nor to describe all currently available different electronic nose technologies, for which other extensive review papers already exist [4,15,16,48,49], but it is limited to those works proving the evolution of such instruments from research objects to regulatory tools.

The second part (Section 3 and Section 4) reviews the relevant existing technical standards and guidelines that directly refer to electronic noses or that could possibly be applied to them. In more detail, Section 3 gives a short historical overview of the standardization attempts that have been made in Europe related to electronic noses, although not all necessarily referring exclusively to their environmental applications. Then, Section 4 briefly describes the relevant technical standards referring to other instruments intended for air quality monitoring, i.e., automatic measuring systems (AMSs). Although electronic noses are not AMSs, their implementation for emission and ambient air monitoring purposes, arises the question of the possibility to assimilate them with AMSs

Finally, the last part of this review (Section 5) represents a critical discussion of the possible approaches for the development and the application of standardized quality protocols for electronic noses intended specifically for environmental odour monitoring. In this section, possible alternative or complementary schemes for electronic noses qualification are proposed and discussed. As previously mentioned, such quality protocols are fundamental in order to properly characterize the instruments in terms of performance characteristics, measurement uncertainty, and effective applicability.

It is important to highlight that this paper only focuses on electronic noses used for monitoring odour as a whole, and not for the detection or identification of odorant substances.

## 2. State-of-the-Art of Electronic Nose Applications as Environmental Odour Monitoring Tools

The aim of this section is to give a brief overview of relevant examples of electronic nose applications for environmental odour monitoring, thereby focusing on those works that aimed to promote them as effective air quality monitoring tools. As previously mentioned, this section does not claim to give an exhaustive review of electronic nose applications for environmental odour monitoring, which have been already discussed in a recent review paper [15], but it has the aim only to describe their evolution in time from research objects to potential regulatory tools.

Historically, one of the first important works dealing with the use of an electronic nose for odour analysis and identification was reported by Nicolas et al. in 2000 [50]. This work describes the use of very simple instruments based on SnO_2_ sensors for measurements around real sources of malodour in the environment such as compost facilities, waste water treatment plants, rendering plants, etc. giving promising classification results with discriminant analysis and principal component analysis. The authors also highlight the influence of atmospheric conditions on the sensor responses and the necessity to carry out repeated training over time in order to compensate sensor drift. In a more recent work [51], the same authors describe the application of five electronic noses, each comprising six Metal Oxide Semiconductor (MOS) sensors from Figaro^®^, for the assessment of odour annoyance near a compost facility. The study proves the system to be sufficiently efficient to predict in real time possible odour annoyance in the surroundings of the plant, even though the approach suffers from various uncertainties, from the sensors to the final determination of the distance of downwind annoyance.

One of the first examples in the literature in which the electronic nose is proposed as a methodology to quantitatively determine an odour impact was described by Sironi et al. in 2007 [52]. In this case, two electronic noses, each equipped with six MOS sensors, were used for monitoring odours from a composting plant. After training, one instrument was installed at the plant fence-line, while the second instrument was installed at a receptor located at about 4.3 km from the plant under investigation. The electronic noses analysed the air every 12 min for a 4-day period, then the sensor responses were analysed with the aim of classifying the analysed air into the different olfactory classes considered for training. This way, odour exposure could be assessed in terms of relative recognition frequency of odours from the monitored plant. This study also provides a sort of instrument performance evaluation by comparison of the outputs of the electronic nose installed at the receptor with the recordings of odour episodes of the residents. These data were presented in a confusion matrix and an accuracy index for classification was evaluated, which was equal to 72%. This result was considered as satisfactory, despite the reported influence of atmospheric humidity and sensor drift.

Another study in which the electronic nose responses were evaluated in combination with other sensorial observations (e.g., odour complaints reports and odour observations of experts) was reported by Milan et al. in 2012 [53]. This work describes a huge monitoring program aiming to map the odour impact in the Port of Rotterdam by using 40 fixed and four mobile electronic noses for a 3-year period. The objectives of investigating the electronic nose potential as an odour management tool for reducing odour exposure, as well as a safety management tool for the fast recognition of accidental gases resulting in incidents was considered as promising, although the validation procedure involving the comparison of instrumental and sensorial odour observations was not detailed in this work.

A different interesting application of electronic noses in this field was described by Chirmata et al. in 2015 [54], where electronic noses were used in order to implement a system for the continuous monitoring and tracking of odours in the city of Agadir. In this case, meteorological data were used in order to follow instantly the odour level in the study area and to identify the emission sources.

Finally, a very recent example of electronic nose implementation as an environmental odour monitoring tool in Italy is given by Licen et al. [55], who describes a 4-month survey close to a steel plant in Trieste. In that case, self-organizing maps proved to be a useful tool for visualizing the dynamic evolution of the system with time, thus allowing the identification of possible sources of malodour and evaluate frequency and duration of odour episodes.

Besides these examples, which describe the application of electronic noses as air quality monitoring tools for odour impact assessment, it is worth mentioning here that there are some cases in which the use of electronic noses for odour monitoring is foreseen on a regulatory level.

One first significant example is the French regulation about rendering plants [41], which, in article 46, foresees a reduction of the periodic odour measurement campaigns if a representative and permanent measurement is carried out by means of electronic noses.

Another very interesting example was recently presented by Cangialosi et al. in 2018 [42]. In this case an electronic nose was used in combination with an H_2_S continuous analyser in order to provide a continuous measurement of the odour concentration at the fence-line of a sanitary landfill. An automatic alert to the local authority was set when the odour concentration measured by the electronic nose exceeded 500 ou_E_/m^3^ for more than 5 min or when two consecutive H_2_S concentration measurements at 5 min intervals exceeded 20 ppb. Despite the very interesting application, the results of the study showed that the odour concentration values estimated by the electronic nose were poorly correlated with the H_2_S concentration measurements.

It is worth highlighting that such prescriptions involving the installation of an electronic nose for continuous odour measurement around some plants (and specifically landfills) are now becoming a common trend in the Region of Puglia, in Southern Italy.

## 3. History of Standardization Attempts in the Field of Electronic Olfaction

This section gives a brief historical overview of the standardization attempts that have been made in Europe in the field of electronic olfaction. These do not necessarily refer only to electronic noses for environmental applications.

### 3.1. First Standardization Attempts: The Second Network on Artificial Olfactory Sensing (NOSE II)

As reported in a recent and very interesting opinion paper by T. Nagle and S. Schiffman [43], the first attempt for standardization in the field of electronic olfaction was carried out in 2001, under the sponsorship of the European Commission. The Second Network on Artificial Olfactory Sensing (NOSE II) [56] was constituted and its main task was to work out its own recommendations for standards and to foster their use in the sensor and e-nose community. The work was organized in three working groups (WGs), dealing with the following topics:

- standard data format for electronic nose data
- calibration and standardization
- hardware–software interfaces.

However, in the end, no specific standards were completed. The reasons given were: (i) the large number of available sensor types and electronic nose technologies; (ii) the WG goals were too broad; (iii) failure by the industry to establish a generic electronic nose technology; (iv) no broad industry support for a common data format; and (v) fragmented markets with different application requirements [43].

### 3.2. The NTA 9055.2012

After that, in 2012, the national standardization body of the The Netherlands (NEN) was the first one who succeeded in releasing a technical agreement document (NTA 9055:2012 [57]) regarding the specific use of electronic noses for the monitoring of odour emissions that may cause odour nuisance or safety risks.

As stated in the scope of this document: “the purpose of NTA 9055 is to draw up a list of requirements for using an electronic nose (e-nose) to detect changes in the composition of the ambient air”.

The scope defines the following fields of application:

- Continuous monitoring: since dynamic olfactometry does not allow for continuous odour monitoring, electronic noses can be used for this purpose. It is claimed that “continuous monitoring using e-noses, combined with a knowledge of current process and weather conditions, makes it possible to identify the cause of odour nuisance in a targeted way”.
- Information for risk assessment: electronic noses are proposed as tools enabling quick gathering of information concerning the dispersion of gaseous substances in the case of sudden major emissions, e.g., as a result of an incident. The document affirms that this information could possibly be used as a basis for a risk assessment.
- Emission detection and process monitoring: it is stated that the use of electronic noses for emission detection in industrial applications may help to optimise the process and minimise odour nuisance.

Then, after a brief general description of the electronic nose technology given in Section 1 (and normative references, definitions, and abbreviations reported in Sections 2–4), Section 5 aims to describe the methodology for using an electronic nose. First, the principles of electronic nose training are generically reported. Training involves the recording of the electronic nose signals when exposed to air with a given composition and the correlation of these data with the data acquired by sensory or instrumental analysis of the same air, so that “relevant sensory or instrumental perceptions can be reproduced if the same electronic nose data is recorded”. It is specified that training can be carried out at the measurement site or in a laboratory. In both cases the procedure involves collecting the electronic nose data together with information provided by other means of detection and then correlating the electronic nose data with the other information.

Then, Section 5 of this document gives a general description of electronic nose networks (Section 5.2) and of mobile electronic noses (Section 5.3). Finally, Section 6 very briefly gives some indications about sampling, thereby referring to other existing norms.

Although this technical document has the undeniable value of having been the first technical document published by a standardization body regarding electronic noses, it has the drawback of being extremely synthetic and too generic to achieve the goal of standardization of the procedures for the application of these instruments in the environmental and safety sectors. Despite the statement of the scope of the document, no requirements for the use of electronic noses are defined, except for a generic description of the instrument training. What is totally missing for a standard is a description of the procedures for the verification of the electronic nose functionality nor the validation of the instrument outputs.

After the publication of this first technical document, with all its limitations, the need for standardization on this topic at a European level became evident. For this reason, a few years later, in 2014, the European Committee on Standardization (CEN) promoted the constitution of a working group dedicated to the draft of a standard on instrumental odour monitoring (WG41), whose activity will be detailed in the next section.

### 3.3. The CEN TC/264 WG41 “Instrumental Odour Monitoring”

As previously mentioned, after the publication of the NTA 9055:2012, a new European working group (WG41) was established within CEN TC/264 on air quality to draft a standard related to instrumental odour monitoring systems. The WG41 was composed by experts nominated by national standardization bodies from European countries including Belgium, France, Germany, Italy, The Netherlands, Spain, and the UK [58].

The scope of this standard is to specify requirements for instrumental odour monitoring systems (IOMS) for the monitoring of odour in ambient air and in emissions to ambient air. Indoor air was excluded from the scope of the WG. The primary application is to generate odour metrics that are relevant indicators for the presence and attributes of odour as would be perceived by human observers. A benefit of instrumental odour monitoring systems is that they can be used for continuous measurement [58].

The working item intentionally refers to IOMS and not to electronic noses, in order to include any generic “instrument” that can be applied to the monitoring of environmental odours, independently from its functioning principle or sensing technology. Odour is here considered as a whole, thereby referring to “odour” as the “sensation perceived by means of the olfactory organ in sniffing certain volatile substances”, and not to single odorants. Moreover, given the wide range of different devices for instrumental odour monitoring available on the market, based on a variety of different functional principles for gas sensing, the technical design of such systems, their calibration, training and the mathematical model that is used to convert sensor signals into output metrics are not part of this standard, which considers the system as a “black box”.

According to this approach, the work of the WG was focusing mainly on the validation regimes that can be used to prove performance claims. This included defining specific objectives and limitations, thus establishing procedures aiming to verify the instrument suitability for a specific application and its utilization, within defined boundary conditions.

The validation process consists of comparing the IOMS output metrics with odour assessment metrics obtained with suitable reference methods. The reference methods for odour measurement involve the use of human assessors.

For the task of the identification odour presence and odour classification the reference method is represented by field inspections using panel members according to EN 16841:2016 (part 1 or part 2) [59,60]. The EN 16841:2016 is a European Norm that standardizes the field inspection method for odour assessment in the field by means of qualified panel members. In more detail, part 1 of the standard describes a method (“grid method”) for determining the level of odour exposure within a defined assessment area over a sufficiently long period of time to be representative for the meteorological conditions of that location. Part 2 describes a method (“plume method”) for the determination of the extent of recognizable odours from a specific source using direct observation under specific meteorological conditions.

The reference method for validating the IOMS capability to quantify odours by providing an odour stimulus indicator value is dynamic olfactometry, as described by the EN 13725:2003 [34]. This standard defines the reference method for the determination of the odour concentration of an odorous gas sample using dynamic olfactometry with human assessors, thus providing a common basis for evaluation of odour emissions.

As already mentioned, up to now, the activity of the WG has been focusing more on the instrument final validation than its quality check. The main difficulties related to the development of a European Standard on this topic are associated with the fact that the currently existing technologies and applications in the different European countries, and therefore the expectations of the national representatives in the WG, are sometimes very different from each other.

However, since the activity of the WG is still ongoing and under discussion, it will be not described further in this paper.

### 3.4. The VDI 3518-3:2018

Very recently, in December 2018, the German VDI (Association of German Engineers) published a guideline relating specifically to odour measurements with electronic noses, i.e., the VDI 3518 Part 3 “Multigas sensors—Odour-related measurements with electronic noses and their testing” [61].

This guideline represents an important step forward towards standardization of electronic noses, although it does not refer specifically to the environmental monitoring of odours, but also to other fields of odour measurements. In more detail, the following application categories were identified:

- comfort
- diagnosis
- process monitoring
- safety.

One interesting aspect of the guideline is that it defines three different measurement tasks (functionalities) for electronic noses:

- differentiation, i.e., detecting the presence of odours;
- identification. i.e., determining the odour type;
- quantification, i.e., determining the odour intensity.

As stated in this guideline, since electronic noses can be used for a range of different odour-related measurements, the prerequisite is general suitability for the planned application.

For this reason, the VDI guideline describes a procedure for the instrument verification, which is based on three steps.

First, minimum equipment specifications for electronic noses have to be defined. For the formal testing of the equipment’s suitability, data on usage, on the construction, on operating, and on the basic functionality have to be submitted.

As a second step, metrological functionality of the electronic nose has to be tested. Although demonstrating the metrological functionality of electronic noses is not a definitive proof of their suitability for the specific odour-related application, it has to be considered as a prerequisite for reliable operation of the instrument. If the metrological functionality testing is not passed, then it could hardly be expected that the instrument will be suitable for odour measurement in the desired final application. The test shall prove that, when exposed to a test gas, mechanical effects, electric interference, and climatic ambient factors, the electronic nose outcome shall not be affected by more than a deviation of 30% from the reference value.

Finally, once the metrological suitability of the electronic nose has been demonstrated, further testing is required in order to demonstrate the basic suitability of electronic noses for odour-related measurements. The tests can demonstrate the correlation between the electronic nose outputs and the results of an olfactory (sensorial) odour measurement carried out with human assessors.

Performance testing both for metrological functionality and for suitability for odour measurement has to be carried out with at least three standard test gases, containing odorants with known properties, to be chosen from a list provided in annex B of the guideline. Appropriate tests and odorants are to be chosen and the concrete test conditions agreed in accordance with the intended application.

The principle of the guideline is very interesting, since it tries to adopt a similar verification logic as those applied for other measurement instrumentation, but still considering the peculiarities of the electronic nose with respect to other chemical analysers. However, since the guideline is not application-specific, it does not account for the peculiarities of the single odour-related measurement. As a matter of fact, testing an electronic nose destined to environmental odour monitoring should entail the verification of different requirements as those that are needed, for instance, for medical diagnosis.

As previously mentioned, the guideline fixes minimum performances of the instruments to be guaranteed. The drawback of this approach is that the electronic nose technology is still very “young” and probably not mature enough to have fixed minimum requirements, which might, in the end, represent a limitation for further instrument development. Moreover, the minimum requirements fixed in this guideline are based on industrial certifications, thus hardly applicable to research prototypes and products of small manufacturers.

### 3.5. The Italian UNI1605848 Project

Very recently, in February 2019, the Italian Standardization Body UNI proposed a specific standard on the determination of odours by means of IOMS (instrumental odour monitoring systems) and their qualification. As already mentioned in Section 2, in Italy the use of electronic noses as air quality monitoring tools has grown significantly in the last few years, given that in some specific applications the electronic nose outputs have a legal value. As a consequence, this standard is the expression of an urgent need in Italy to provide the local authorities and the final users with an adequate normative text allowing for qualification of extremely different instruments that are proposed for environmental odour monitoring purposes.

The interesting and innovative thing about this standard is that it defines three quality assurance levels for electronic noses, following the principles of the EN 14181:2014 referred to automatic measuring systems (AMSs) for the continuous monitoring of emissions and ambient air, which will be described in the next section.

As a first step, electronic noses for environmental odour monitoring should undergo an initial metrological verification. This verification should be carried out by the manufacturer, in order to declare the instrument properties before its installation in the field. The parameters that shall be defined are, among others, the expected sensor responses to reference gases (whose composition is not specified and shall be chosen by the manufacturer) at zero and span, the effect of temperature and humidity on the sensor responses, and the response time T90. By definition, T90 is the time needed for a detector to measure 90% of the applied concentration level. In the case of electronic noses, T90 would be the time needed for the sensor to reach 90% of its maximum response to the applied reference gas.

It is also required that the capability of the instrument to provide correct results coherently with the type of determination (i.e., odour presence, odour class, or odour quantity) is verified in the laboratory before its installation in the field. The standard does not specify how this should be done in detail; the manufacturer shall provide all information related to the metrological verifications in a specific report that describes the methodology adopted.

The usefulness of this information is that, during its application in the field, the electronic nose functionality can be tested periodically by verifying that its properties declared in this initial metrological verification are still satisfied.

The essential verification step is carried out after installation in the field in order to verify the IOMS functionality as a whole, thereby including training, according to the reference method EN 13725:2003. The procedures for the verification depend on the type of determination requested (i.e., odour presence, odour class, or odour quantity). For any type of determination, the verification shall involve the comparison of at least 15 measurements provided by the electronic nose and 15 simultaneous measurements conducted in conformity with the EN 13725:2003, in the conditions that are considered to be most representative of the application. Measurements shall be carried out on at least four different days and be distributed in at least 6 h for each test day, in order to cover a 24-h cycle. The standard does not describe how the verification shall be carried out in detail, but it is required that a verification report is produced reporting all the relevant information about the test methods and results.

A mathematical approach is proposed for the evaluation of the deviation of the odour quantities provided by the electronic nose from the odour concentrations measured by dynamic olfactometry according to EN 13725:2003.

This standard, despite not being very detailed about the testing procedures, has the big advantage of being the only approach that is based on a multi-level verification of the electronic nose, which is fundamental in order to enable the instrument qualification in every phase of its life as an air quality monitoring tool.

## 4. Relevant Technical Norms Related to Other Instruments for Air Quality Monitoring

Besides the technical documents or standardization attempts described above, which are related directly to electronic noses, but not necessarily to air quality monitoring, other technical norms exist that refer to other instrumentation for air quality monitoring, which can be considered as inspiring for the development of quality protocols for electronic noses to be used in the field of environmental odour monitoring.

This is the case for the so called “automatic measuring systems” (AMSs), which are automatic analysers used for the continuous monitoring either of emissions or of ambient air.

Starting from 2000, CEN experts made a great effort to define all the aspects of AMSs. In more detail, the two documents that define the basic structure of AMSs are the standards EN 14181:2014 (first edition was released in 2005) and the EN 15267 series [44]. These technical standards, which describe the quality programs that must be followed in the realisation, validation and management of an AMS, will be briefly described in this section; then their applicability to electronic noses will be discussed in the next section, dedicated to the description of the approaches that can be adopted for the development of quality protocols for instrumental odour monitoring systems.

### 4.1. The EN 14181:2014

The Standard EN 14181:2014 [62] specifies the procedures for establishing quality assurance levels (QALs) for AMS installed on stationary sources for the determination of the flue gas components and other parameters. The following levels are defined [44].

- QAL1: the AMS, intended as the entire system—from the sampling up to the final result output—has to fulfil both general requirements (e.g., quality and safety requirements, availability, stability, sensitivity) as well as specific requirements related to the application (e.g., matrix of flue gas, interferents, emission limits, type of installation, weather conditions). For this purpose, a complete evaluation of the expected performance shall be carried out based on a detailed engineering project of the system, thereby using the mathematical formulations given by the Standard. The QAL1 process is considered complete when, based on the AMS design, it is possible to ensure that the uncertainty of the AMS is always below a given value.
- QAL2: QAL2 entails the initial technical verification of the system hardware and software after installation, in order to verify both the compliance with the design and especially the calibration of the system. QAL 2 verifications shall ensure that AMS measurements are reliable with the relevant standard reference method (SRM). For every pollutant or chemical compound of interest, the relevant SRM represents the only “legally binding” method for the limit verification of that compound.
- QAL3: If QAL2 is accomplished, the AMS enters into “normal service”. After that, the QAL3 procedure involves the establishment of a continuous Quality Assurance (QA)/Quality Control (QC) plan, in order to guarantee that the AMS is fully operative over time.
- Annual surveillance test (AST): every year, an independent verification test is required to check the AMS operation in order to verify QA/QC and maintenance procedure, or to solve the non-conformities eventually raised.

### 4.2. The EN 15267-1/2/3:2009

EN 15267-1/2/3:2009 [63,64,65] specifies the general principles, including common procedures and requirements, for the product certification of AMSs for monitoring ambient air quality and emissions from stationary sources. This product certification consists of the following sequential stages [44]:

- performance testing of an automated measuring system;
- initial assessment of the AMS manufacturer’s quality management system;
- certification;
- surveillance.

In more detail, the scope of EN 15267 Standards series is:

- The specification of requirements for the manufacturer’s quality management system, the initial assessment of the manufacturer’s production control and the continuing surveillance of the effect of subsequent design changes on the performance of the AMS. It also serves as a reference document for auditing the manufacturer’s quality management system.
- The definition of the performance criteria and test procedures for the AMS. It provides the detailed procedures covering the QAL1 requirements of the EN 14181:2014 and, where required, the input data to be used in QAL3.

This European Standard applies to the certification of all AMS for monitoring ambient air quality and emissions from stationary sources for which performance criteria and test procedures are available as European Standard.

## 5. Approaches for the Development of Quality Protocols for Instrumental Odour Monitoring Systems

For the specific application of electronic noses to environmental odour monitoring, different approaches could possibly be adopted for the development of procedures for the instrument qualification. These approaches are discussed in this section.

### 5.1. Approaches for the Qualification of Electronic Noses Described in the Scientific Literature

Other more or less similar approaches for e-nose performance testing have been proposed in the scientific literature. An example of approach that can be used for qualification of electronic noses to be used as environmental odour monitoring systems is given in References [24,66].

In more detail, the first example [24] proposes a testing procedure for performance evaluation aimed at the definition of minimum performance requirements referred to electronic noses to be used for environmental odour monitoring at receptors.

In this study, the following aspects were deemed important for qualifying an electronic nose for environmental odour monitoring:

- the capability of giving repeatable and reliable responses under variable atmospheric conditions; indeed, variations of temperature and humidity are particularly critical for e-nose environmental outdoor use (“invariability of responses to variable atmospheric conditions”);
- the sensitivity to odours: if e-noses are used at receptors they are likely to be exposed to very diluted concentrations, for this reason, instruments shall have a very high sensitivity (i.e., a low “detection limit”);
- the capability of correctly classifying the detected odours, by recognizing their provenance and attributing them to the correct olfactory class (“classification accuracy”).

Then the study describes as example of detailed procedure adopted to test a commercial electronic nose towards those aspects. According to the proposed procedure, the abovementioned aspects shall be tested using standard test gases. The use of standard test gases is preferred over “real” environmental samples, because of the intention of guaranteeing repeatability and reproducibility of the procedure, in order to make it possible to compare the performances of different instruments tested in different labs or at different times and conditions.

The second example [66] proposes a procedure for electronic nose performance testing based on a similar approach. In this case, the instrument performances are evaluated through the definition of an experimental protocol, which is structured into different levels.

The paper focuses on the first two levels of testing, which involve specific tests with standard test gases, including for instance chemical compounds that are representative of common environmental odours. These specific tests allow for the evaluation of the electronic nose limit of detection, lower detection limit towards those target compounds and repeatability of responses to a given stimulus. This type of testing is not related to specific applications and therefore raw signals are considered instead of instrument output for performance evaluations. On the other hand, the third level of testing is related to the specific application, and thus the performance of the trained system is evaluated in the field in terms of classification accuracy. This third level of testing was not described in the paper [66].

The two abovementioned papers have in common that the proposed approach is based on the idea of verifying some fundamental aspects (e.g., lower detection limit, response repeatability, capability of compensating humidity and temperature variations, and capability to classify different odour types correctly) by performing performance testing using standard test gases.

It is important to highlight that since standard test gases are never fully representative of what happens in “real” environmental conditions, these tests shall not be considered as a sufficient condition to prove the electronic nose suitability for the specific application. What is described here is a sort of pre-qualification, which always needs to be followed by a validation in the field.

This is not very dissimilar from the approach proposed in the VDI 3518-3:2018; the main difference is that here no minimum performance requirements are fixed.

### 5.2. Applicability of EN 14181:2014 and EN 15267:2009 to Electronic Noses

#### 5.2.1. Electronic Nose as AMS?

As described in the previous section, the Technical Standards EN 14181:2014 and EN 15267:2009 define the quality protocols that must be applied to AMS, which in facts are systems for the continuous sampling, measurement and control of pollutants in emissions and ambient air.

In a very recent paper by Cipriano [44], the author proposed the possibility of implementing the features of the technical standards for AMSs to electronic noses, thereby focusing on qualification and maintenance, and on the uncertainty aspects.

Although electronic noses are not AMSs, and thus this proposal may sound as a provocation, the implementation of electronic noses for emission and ambient air monitoring purposes, as described in the previous section, the question arises of the opportunity to partially assimilate them to AMSs. A possible integration of such instruments into the universe of AMSs could be advantageous in order to define the uncertainties associated with their use as air quality monitoring tools.

Indeed, an AMS device is intended to be used for continuous legal use, so it must ensure reliability, integrity and data security. It shall allow the calculation of both the uncertainty on the measured values vs. a SRM, and an independent verification of its metrological capabilities.

The possibility to apply the principles of the standards for AMSs to the verification of these same aspects is discussed here, as proposed in Reference [44].

#### 5.2.2. Reliability, Integrity and Data Security

**Figure 1:**
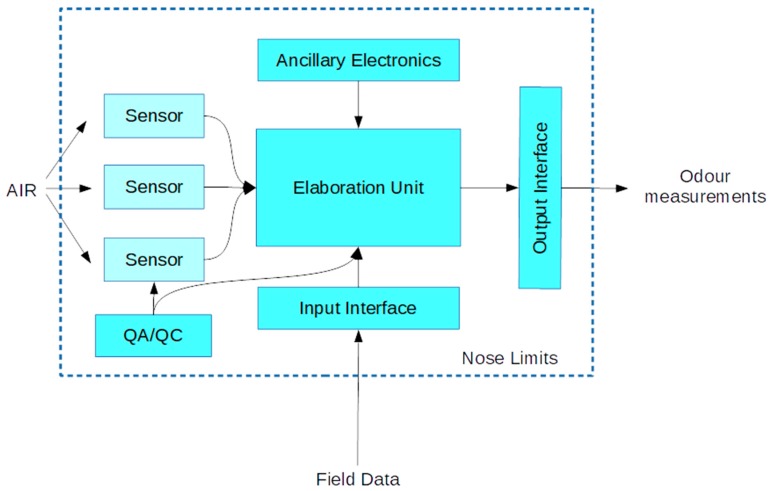
Electronic nose with quality assurance (QA)/QC control of the sensor array.

In order to allow QAL1 and QAL2 evaluation, electronic noses shall declare the performances of the entire system (sensors, data acquisition, processing, interfaces, etc.) and permit their verification. Also, in order to allow QAL3 procedures, it is necessary to have specific hardware and software solutions to implement periodic checks of the sensors array (Figure 1).

One of the biggest advantages of electronic noses is their flexibility due to possible modification of the software, by using different algorithms and calibration data. Such flexibility can be very useful for obtaining a good QAL2, so that the instrument can be trained to give results very close to the calibration values. However, this is also one of the biggest problems during ongoing verifications, as every modification done after QAL2 process implies the formal invalidation of its performances. E-nose structure shall ensure that, when the calibration process is finished, all the relevant data and configurations are locked and encrypted in order to prevent performances changes and unauthorized modifications.

#### 5.2.3. Uncertainty

Nowadays all emission and ambient air monitors shall declare their “uncertainty budget” evaluated following principles of the ISO IEC Guide 98-3:2008 “Guide to expression of uncertainty in measurement” [67]. In this guide, the output of the measuring process is described by Equation (1) [44]:

(1) $$y=f\left(x_{1},x_{2},\ldots ,x_{n}\right).$$

The estimation of the total uncertainty u(y) can be obtained by the propagation of the single uncertainty terms for each *x_i_*, *u(x_i_)*, by the means of Equation (2):

(2) $$u\left(y\right)=\sqrt{\sum C_{i}^{2}u^{2}\left(x_{i}\right)},$$

where *C_i_* is the sensitivity coefficient of the single *x_i_* term, and *u(x_i_)* its uncertainty.

Function *f* shall cover all the measuring process, from the single sensor acquisition up to the final output, including calibrations, interferences, nonlinearities and software-related errors. For that, the use of a new CEN standard, the FprCEN/TS 17198:2017 “Stationary source emissions—Predictive Emission Monitoring Systems (PEMS)—Applicability, execution and quality assurance” could be useful. Such a standard is designed for emission prediction models, but could be easily adapted to electronic noses. Furthermore, its scope is to achieve conformity to EN 14181 and EN 15267 of software predictive systems and furnishes a simplified formula for uncertainty evaluation [44]:

(3) $$u\left(y\right)=\sqrt{\left(u_{model}^{2}+u_{input}^{2}+u_{other}^{2}\right)},$$

where *u_model_* is the uncertainty of the mathematical model, *u_input_* the uncertainty from sensors array, *u_other_* the uncertainty due to parameters not included in the model, evaluated by confrontation with independent odour measurements used to calibrate the electronic nose.

#### 5.2.4. Independent Verifications

An e-nose shall make it possible for the final user to verify its metrological capabilities, in order to validate its operational status and guarantee its calibrations are still aligned with QAL2 results. This means that, for instance, a specific gas matrix should be realised and used for periodic system validation.

## 6. Discussion and Conclusions

As stated in the introduction, this paper had the primary aim of discussing the need for the definition of quality protocols for electronic noses that are intended as air quality monitoring tools for the detection and the measurement of environmental odours. As a part of this discussion, this work also described some of the possible approaches that can be adopted for a standardization and qualification of such instruments, thereby reviewing the relevant documents—technical standards and scientific publications—on the issue.

In order to allow a better evaluation of these qualification approaches in terms of practical applicability to e-noses for environmental use, as well as the possible advantages and drawbacks related to their application, Table 1 schematizes the most relevant aspects relevant to the different approaches, together with their pros and cons.

Based on the information reported in Table 1, it is possible to appreciate the pros and cons of the different approaches. Leaving out the Dutch NTA 9055:2012, which is so generic that no real standardization/qualification approach is proposed, and the CEN TC/264 WG41 activity, which is still in progress and at a very preliminary stage, it is possible to make some more specific considerations about the other technical documents reviewed here.

The qualification approach based on the combination of the EN 14181:2014 and the EN 15267:2009 has the enormous advantage, compared to the others, that it is the only one validated. However, despite being a consolidated and effective approach, it is only valid (and validated) for monitoring systems that are not e-noses, and thus would need to be deeply re-adapted in order to make it applicable to odour measurement systems.

On the other hand, the other approaches, which are specific for e-noses and for odour measurement, to the best of our knowledge, are not validated yet. Therefore, up to now, there is no available data comparing how different instruments would perform by application of any of these testing protocols. The only data available in literature concern testing procedures that are not part of a standard [66,67].

One aspect that emerges from the information reported in Table 1, is that two different concepts for standardization/qualification can be distinguished. The first one involves the definition of minimum performance requirements. Even though the establishment of minimum requirements may result in a higher level of guarantee for the end user, this may entail the drawback of being a bit premature for a technology that is still under development. This is particularly true in consideration that e-noses for environmental use can be applied in a variety of different situations and with different purposes, for which different instrumental performance characteristics may be required. For this reason, it might be better to have a more flexible approach involving instrument testing and performance declaration without the necessity to achieve a minimum performance level.

Thus, a possible alternative approach for a quality protocol regarding electronic noses to be used for environmental odour monitoring would be a combination of the concept of performance testing with the requirements of the quality standards for AMS. This would entail a method for performance testing and ongoing quality procedures (QAL3), based on the principle that the instrument manufacturer shall specify the electronic nose characteristics, metrological functionality, and design limits, without fixing minimum performances. Thus, it is the final user who evaluates the instrument suitability and makes the choice of the most appropriate electronic nose features according to his specific needs, or, for instance, to the current regulatory requirements.

This is basically the approach followed by the Italian UNI1605848 project, which, despite its lack of details regarding the testing procedure, is the only multi-level verification approach allowing for the characterization of the different aspects of the electronic nose functioning in every phase of the instrument’s life and application.

This approach seems to guarantee higher flexibility, thus making it applicable also to prototypal solutions, which is an important aspect for a technology that is still at an initial stage as proper air quality monitoring tool and in continuous development.

As a conclusion, application of specific quality programs to electronic noses for air quality monitoring is a delicate and complicated process, not yet deeply explored, and where there are continuous implementations of both the instruments and the relevant technical standardisation. However, the current status of the technology seems to be sufficiently mature to undergo such a process, which is necessary in order to make electronic noses a widespread and effective environmental odour impact assessment tool.

## Figures and Tables

**Table 1:** Schematization of the existing approaches that can be applied for e-nose qualification and standardization in environmental odour monitoring applications.

Approach	Nationality	Specific for E-Nose?	Specific for Environmental Use?	Principle of Standardization	Pros	Cons
NTA 9055:2012	The Netherlands	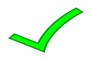	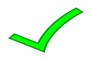	None	Historical value: it is the first national technical document on e-noses	Too generic Specific testing procedures are not defined No standardization approach is proposed
EN14181:2014 + EN15267:2009	Europe	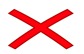	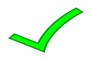	Performance declaration	Complete and validated approach (for AMS) Stepwise approach with three different testing levels for performance verification Allows comparison of systems based on different functioning principles	Not referred to e-noses Would need to be revised to make it applicable to odour measurement systems
VDI 3518-3:2018	Germany	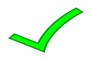	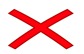	Minimum requirements	Very complete and detailed guideline Specific for different e-nose applications Identification of three different tasks (i.e., differentiation, identification and quantification), each involving specific testing procedures Two-levels testing: (1) metrological functionality, and (2) suitability for odour measurement	Measurement uncertainty is mentioned, but a method for evaluation of this uncertainty is not specified Minimum requirements are fixed, and are mostly based on industrial certifications: this might be premature for a technology that is still under development, and consequently limit the development of research prototypes
CEN TC/264 WG41	Europe	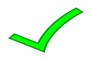	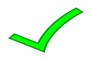	Not defined yet	Standardization attempt on an international level: the WG includes experts from several European countries Identification of three different tasks (i.e., differentiation, identification and quantification), each involving specific testing procedures “Black-box” approach	Still in progress, at a very preliminary stage Not a stepwise approach: only focused on final validation Does not allow comparison of different systems prior to their installation in the field Not validated
UNI 1605848	Italy	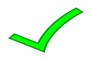	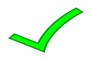	Performance declaration	Stepwise testing procedure inspired to the QALs of the EN 14181:2014 for AMS Three types of possible determinations are defined (i.e., odour presence, odour class, or odour quantity), each involving specific qualification procedures A method for the measurement uncertainty evaluation is proposed Flexible approach: testing procedures are not fixed, but they shall be detailed together with the achieved performances in a specific report This approach leaves some freedom in the characterization of instruments and in the development of new technologies	Definition of the instrument pre-qualification testing procedures are lacking The procedures for comparison with reference methods (e.g., EN 13725:2003) are not defined in detail for each type of determination Not validated yet
